# Mesoscopic Mechanical Properties of Aggregate Structure in Asphalt Mixtures and Gradation Optimization

**DOI:** 10.3390/ma16134709

**Published:** 2023-06-29

**Authors:** Jingchun Chen, Jian Wang, Min Li, Zedong Zhao, Jiaolong Ren

**Affiliations:** 1 School of Civil Engineering and Geomatics, Shandong University of Technology, Zibo 255000, China; 2 School of Transportation and Vehicle Engineering, Shandong University of Technology, Zibo 255000, China

**Keywords:** coarse aggregate structure, DEM, mechanical properties, gradation optimization, interlocking force, contact force

## Abstract

Particle media are widely used in engineering and greatly influence the performance of engineering materials. Asphalt mixtures are multi-phase composite materials, of which coarse aggregates account for more than 60%. These coarse aggregates form a stable structure to transfer and disperse traffic loads. Therefore, knowing how to adjust the structural composition of coarse aggregates to optimize their performance is the key to optimize the performance of asphalt mixtures. In this study, the effects of different roughness and different sizes on the interlocking force and contact force of coarse aggregates were investigated through means of simulation (DEM), and then the formation-evolution mechanism of the coarse aggregate structure and the role of different sizes of aggregates in the coarse aggregate structure were analyzed. Subsequently, the optimal ratio of coarse aggregates was explored through indoor tests, and finally, the gradation of asphalt mixture based on the optimization of fine structure was formed and verified through indoor tests. The results showed that the major model can effectively reveal the role of different types of aggregates in the fine structure and the relationship between the strength of contact forces between them and clarify that the strength of the fine structure increases with the increase in aggregate roughness. Hence, the coarse aggregate structure can be regarded as a contact force transmission system composed of some strong and sub-strong contact forces. Their formation-evolution mechanism can be regarded as a process of the formation of strong and sub-strong contact forces and the transformation from sub-strong contact force to strong contact force. Moreover, the dynamic stability of the optimized graded asphalt mixture was increased by 30%, and the fracture toughness was increased by 26%.

## 1. Introduction

As the most widely used road pavement materials, the asphalt mixture plays an important role in infrastructure construction. In the asphalt mixture as an asphalt–void–aggregate composition of multi-phase complex materials (particulate materials), its coarse aggregates account for more than 60%, and these coarse aggregates can form a stable structure to transfer and disperse the traffic load. Therefore, there is a significant correlation between coarse aggregate and the performance of the asphalt mixture. Shen et al. [1,2] specifically pointed out that the coarse aggregate structure is the most critical factor affecting the performance of asphalt mixtures, which was also proved by Pouranian [3] and Lira et al. [4] In addition, existing studies have shown that the key factor affecting the aggregate is not the number of contact points but the number and distribution of strong contact forces. Hence, it is reasonable to believe that the aggregate structure can be optimized more effectively from the perspective of mechanical properties.

Shashidhar et al. [5] applied an image approach to analyzing the contact force transmission paths of the aggregate structure. Sun et al. [6] studied the effect of coarse aggregate meso-structure on the characteristics of the asphalt mixture through the technology of CT and DCT. However, the complexity and uncertainty of the asphalt mixture materials could not be fundamentally revealed only through conventional macroscopic tests. Therefore, more and more studies [7,8] analyze the asphalt mixtures from a mesoscopic perspective and try to establish effective relationships between mesoscopic properties and macroscopic behavior. Ren et al. [9,10] revealed the effect of different void ratios and void sizes on the fracture behavior of the asphalt mixture. Liu et al. [11] discussed the effect of particle size and specimen scale on the crack resistance behavior of asphalt mixtures. Wang et al. [12] constructed a model by DEM to analyze the deformation mechanism of the asphalt mixture during the loading test from the fine view level. Unfortunately, existing studies have explored the variation of properties more through material properties and proportions but not from the perspective of aggregate structure.

Currently, only a few studies have analyzed the structural mechanical properties of aggregates. Cal et al. [13] established a model to quantify the effect of gradation and estimated the shear modulus of asphalt materials. Chen et al. [14] revealed the properties of asphalt mixtures with different sizes of aggregate particles and different gradations based on DEM simulation. Pouranian et al. [15] established a new framework to study packing behavior for the asphalt mixture. All these studies help to deepen our understanding of the structural mechanical properties of aggregates. However, due to the complexity of the aggregate contact structure, the analysis of the mechanical properties of the aggregate structure is not a simple task. Some shortcomings of the existing studies are as follows: In road projects, aggregates are divided into many different sizes according to their particle size, and the ratio of different sizes of aggregates greatly affects the mechanical properties of road materials. However, the existing studies have neglected to investigate the effect on aggregate gradation. In addition, the formation-evolution mechanism of the aggregate structure has not been revealed.

Hence, this study analyzed the macro-skeleton interlocking forces and meso-aggregate contact force of the coarse aggregate structure based on the DEM simulation and clarified the formation–evolution mechanism and the function of different types of aggregates on the coarse aggregate structure. On this basis, the coarse aggregate structure is optimized based on the principle of optimal aggregate skeleton strength, and a better asphalt mixture gradation is proposed, which passed the indoor test verification.

## 2. Materials

### 2.1. Asphalt

In this paper, AH-70 asphalt is adopted, and the basic performance of AH-70 asphalt is tested according to the specification [16]. Its indexes meet the requirements shown in Table 1.

### 2.2. Aggregate

The physical and mechanical properties of the aggregate affect the road characteristics of the asphalt mixture. Coarse aggregate is mainly used to form the skeleton structure, fine aggregate is mainly used to fill the skeleton structure of coarse aggregate, mineral powder and asphalt to form asphalt slurry filled in the gaps between coarse and fine aggregate and the aggregate bonded together, so that the asphalt mixture to produce the ability to resist the traffic load. The aggregate is obtained from limestone in Shandong Province, China. The test results [17] of the basic characteristics of the aggregates are shown in Table 2.

## 3. Numerical Model

### 3.1. Numerical Model

Due to the particularity of DEM numerical calculation, the complexity of model operation depends on the number of particles and contact points. The DEM simulation of unbonded particles is usually random, and these particles need to reach the equilibrium state of contact force under gravity before the calculation begins. However, the existing numerical models [18,19,20] involve a large number of particles, so it takes a lot of time to find the equilibrium state of contact force. Therefore, a new simplified modeling method is adopted to study the mechanical properties of aggregate structures by referring to the experience of existing studies [21,22]. The specific process is as follows:

The two-dimensional mapping area (*S_i_*) of coarse aggregate is calculated according to the mass, apparent density and size of different coarse aggregates, as shown in Equation (1). Then, the built-in command “ball” of PFC^2D^ is used to generate circular particles in the simulated cylinder and make them meet the particle size requirements of a coarse aggregate.

(1) $$S_{i}=\frac{4m_{i}}{\pi \rho_{i}W}$$

**Figure 1:**
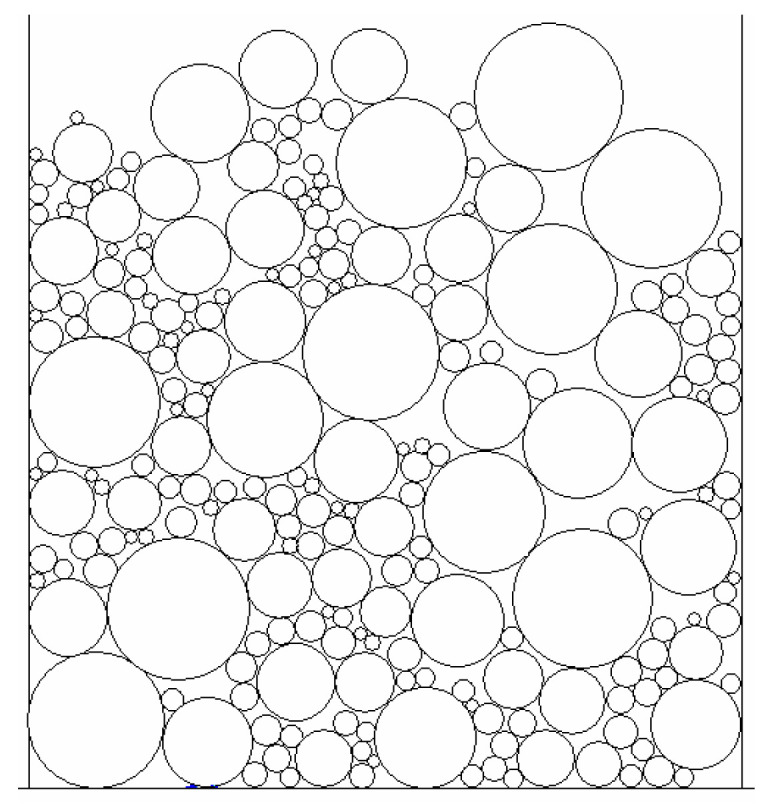
The initial model of aggregate structure.

The initial model is generated by combing different aggregates with particle sizes including 13.2~19 mm, 9.5~13.2 mm, 4.75~9.5 mm and 2.36~4.75 mm, as shown in Figure 1.

**Figure 2:**
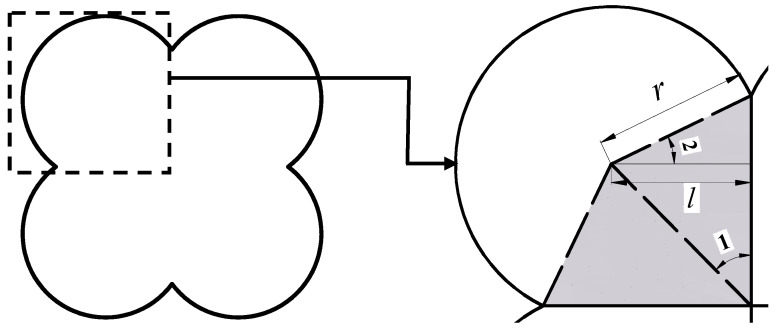
The structure of irregular particles.

Secondly, the built-in command “b_id” of PFC^2D^ is used to automatically search the particles numbered t in the *i*-th type coarse aggregate and read their center coordinates, radius and so on. Moreover, according to the surface non-uniformity coefficient (*a*) of the coarse aggregate, four sub-particles are generated by the built-in command “ball” of PFC^2D^. The structure of irregular particles is shown in Figure 2.

As shown in Figure 2, the area of irregular aggregate (*S*) can be represented by Equation (2). Then, *S* is set qual to the initial circular particle area (*S_o_*), as shown in Equation (3). On this basis, the radius of the particle is obtained by Equation (4).

(2) $$S=4r_{it}^{2}[a\sqrt{1-a^{2}}+a^{2}/\tan(\pi /4)+3\pi /4-\arccos a]$$

(3) $$S=S_{o}=\pi R_{it}^{2}$$

(4) $$r_{it}=\sqrt{\frac{\pi R_{it}^{2}}{4[a\sqrt{1-a^{2}}+a^{2}/\tan(\pi /4)+3\pi /4-\arccos a]}}$$

Subsequently, the center coordinates of the four small particles are calculated according to Equations (5)–(8).

(5) $$x_{1,it}=X_{it}-ar_{it}$$

(6) $$x_{2,it}=X_{it}+ar_{it}$$

(7) $$y_{1,it}=Y_{it}-ar_{it}$$

(8) $$y_{2,it}=Y_{it}+ar_{it}$$

**Figure 3:**
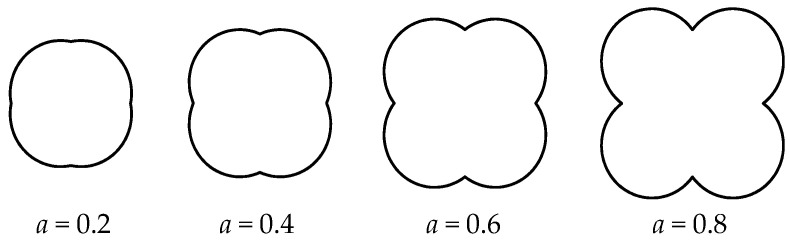
The irregular aggregate of different *a* values.

A series of particles of different *a* values are shown in Figure 3. The greater roughness of the aggregate is seen obviously with the larger *a* value.

**Figure 4:**
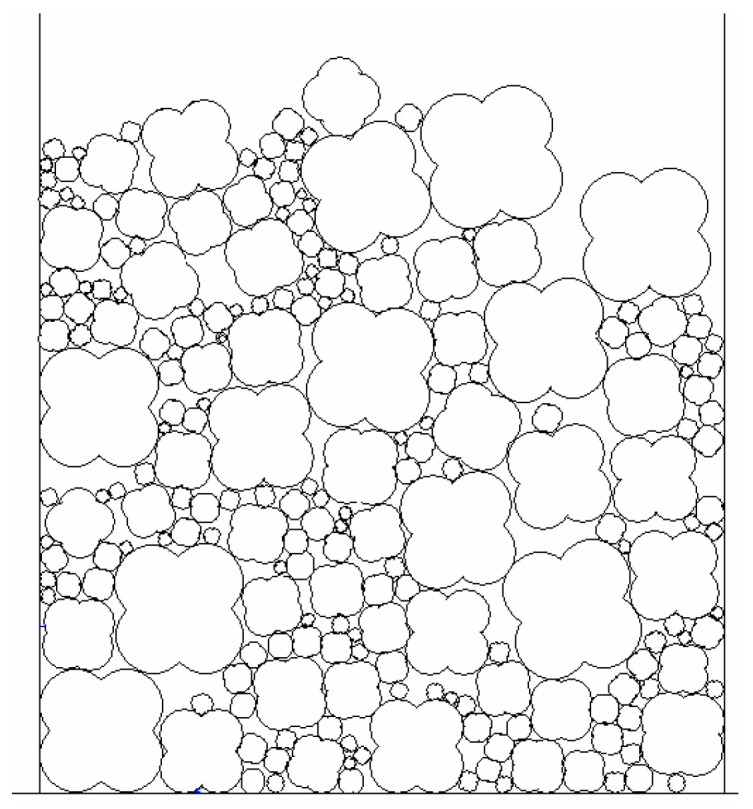
The numerical model of irregular particle structure.

In the end, the irregular particles are composed of four sub-particles by using “clump” in the PFC^2D^. Then, initial particles of number t are removed by using “delete” in the PFC^2D^ and replaced by the corresponding irregular particles. When the aggregate structure reaches the static equilibrium state under the action of gravity, the numerical model of the irregular particle structure can be obtained, as shown in Figure 4.

In addition, the simplified Hertz–Mindlin constitutive is adopted because it can better describe the nonlinear characteristics of aggregate structures, as shown in Equations (9)–(11).

(9) $$K_{n}=\frac{2}{3}\frac{G\sqrt{2U\bar{R}}}{1-\varepsilon }$$

(10) $$K_{s}={\frac{2\left[3G^{2}\left(1-\varepsilon \right)\bar{R}\right]}{2-\varepsilon }}^{1/3}{\left|f_{n}\right|}^{1/3}$$

(11) $$f_{s}=\mu \left|f_{n}\right|$$

### 3.2. Numerical Test and Verification

#### 3.2.1. Numerical Tests

**Figure 5:**
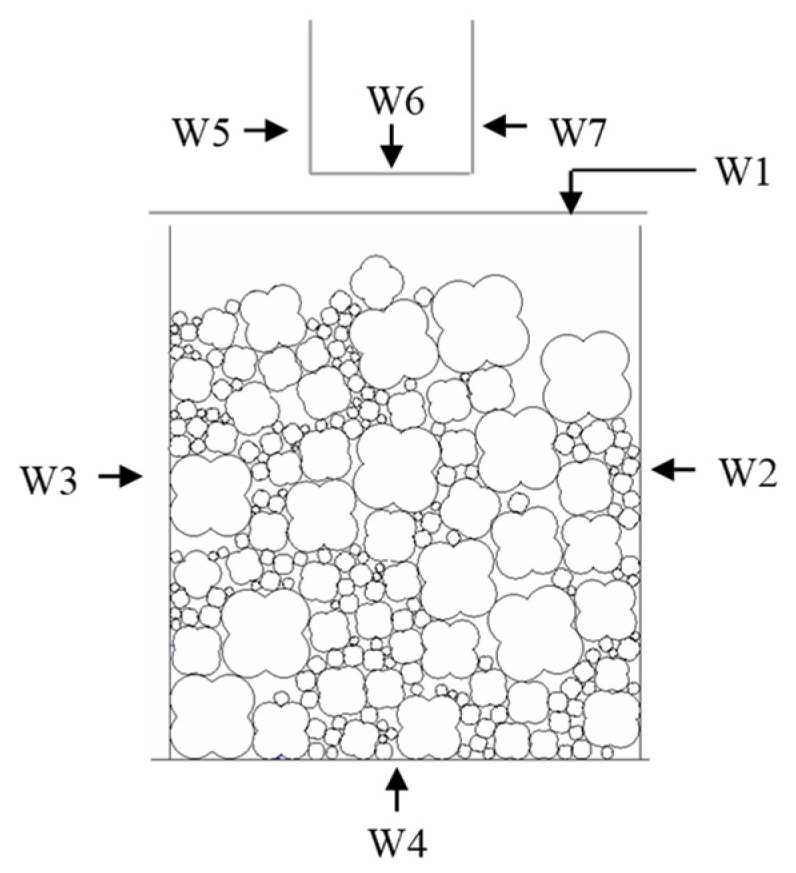
Numerical sample.

According to Section 2.1, numerical sample is randomly generated in a square area, as shown in Figure 5.

In order to simulate the confining pressure effect in the test, the speed of walls (W1, W2, W3 and W4) are controlled by the servo mechanism. When the aggregate structure is compacted to reach the target porosity, the servo mechanism of the W1 and W4 stops running, while that of the W2 and W3 keeps running during the whole numerical test. The servo mechanism can be listed as follows:

(12) $$v^{t+1}-v^{t}=\frac{\Delta \sigma \alpha l}{k_{n}N}$$

**Figure 6:**
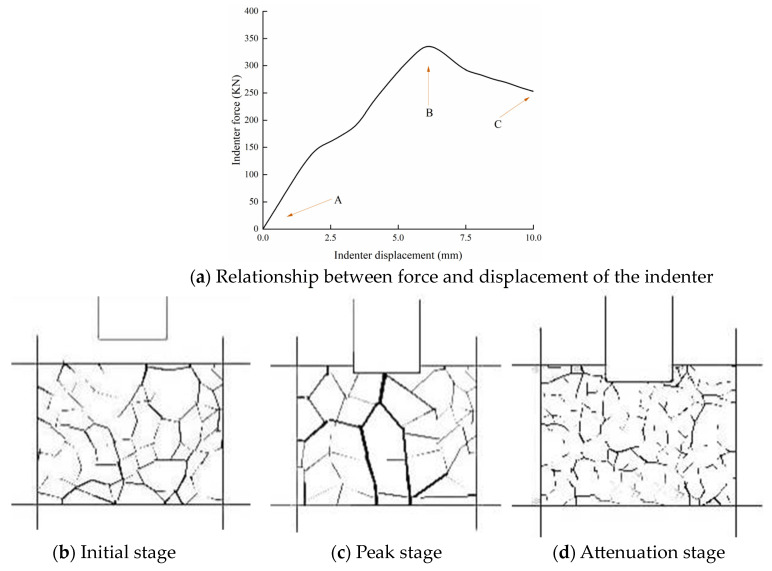
Numerical simulation results.

The simulated indenter is penetrated into the numerical model of aggregate structure at a constant speed, and the contact force and displacement of the indenter are recorded during the whole simulated penetration process. The numerical simulation results are shown in Figure 6. The experiment is a reference specification [23]. The loading speed of both virtual and indoor tests in this study is 1 mm/min for a better comparison.

(1) The relationship between force and displacement of the indenter

Figure 6a shows the relationship between force and displacement of the indenter, where the peak of indenter force is the interlocking force of aggregate structure. It can be found obviously that there are two stages: A-B stage is a stable increase, while B-C stage is the opposite. The evolution process of aggregate contact force in different stages is revealed further in the Figure 6b–d. The black network is the mesoscopic contact force between the particles, and the darker the color, the greater the value of the contact force.

(2) Initial stage

It can be seen that the particle contact force is uniformly distributed in Figure 6b. When the simulated indenter began to penetrate into the numerical model of aggregate structure, the particle contact force gradually increased to resist the penetration of the indenter, which corresponds to the curve state of A-B.

(3) Peak stage

The particle contact force is not uniformly distributed under the action of load and has an obvious phenomenon of concentration of contact force, as shown in Figure 6c. The particle contact force network composed of a series of robust particle contact forces in the red circle can be regarded as a representation of aggregate structure. In other words, the aggregate structure can be considered as a contact force transmission structure composed of a series of strong particles. With the increase in head displacement, the contact force will reach the peak point (B), which indicates that the aggregate structure reached the ultimate bearing capacity.

(4) Attenuation stage

Subsequently, when the contact force of the indenter exceeds the peak value, the aggregate structure begins to collapse, which corresponds to the curve state of B-C. Correspondingly, in Figure 6d, there is no significant contact force of coarse particles.

#### 3.2.2. Results Verification

**Figure 7:**
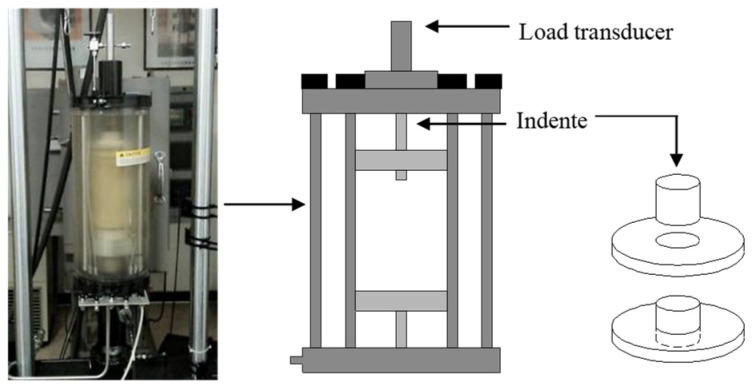
Test device.

Based on the conventional triaxial test system, this paper reproduces the above numerical simulation loading process by setting an improved loading head to measure the interlocking forces of the aggregate structure. The schematic diagram of the test device is shown in Figure 7.

**Figure 8:**
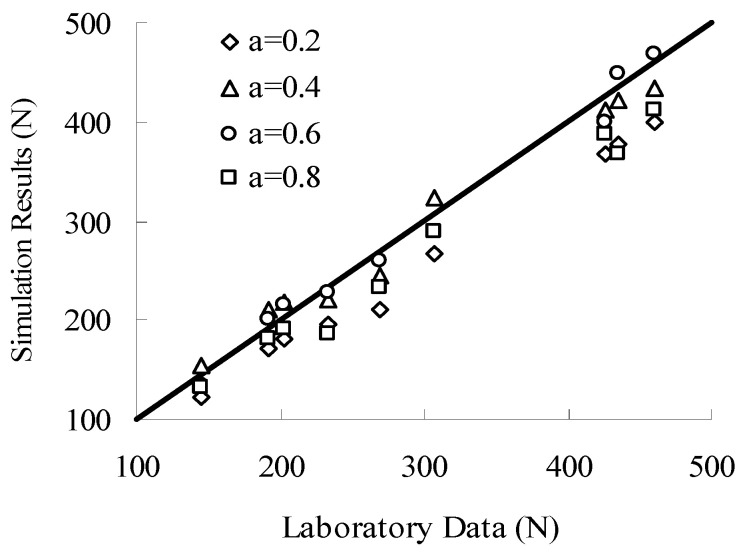
Comparison of simulation results and test results.

The interlocking forces of the aggregate structure are evaluated by using a series of laboratory and numerical tests. As shown in Equations (9)–(11), three DEM contact parameters (shear modulus, Poisson’s ratio and friction coefficient) need to be assigned to the numerical model of aggregate structure. The shear modulus and Poisson’s ratio can be measured by corresponding tests. However, the friction coefficient is obtained through the indoor test inversion after the change in *a* value and follows the principle of the highest simulation accuracy. The comparison of simulation results and test results of aggregate structures with different *a* values is shown in Figure 8.

As shown in Figure 8, the simulation results (*a* = 0.6) have the best accuracy, with an average error of only 4.9%. Hence, the parameters corresponding to *a* = 0.6 (shear modulus of 21.5 GPa, Poisson’s ratio of 0.3 and friction coefficient of 0.5) are used in this section for the later numerical simulations. In addition, the same parameters are used for other *a* value to facilitate the comparison between the simulation results.

However, the above simulations are also flawed in that 2D aggregate structural bodies are referenced instead of 3D structural bodies, which are known to affect the contact force paths. Moreover, 3D model has the problems of long simulation time and high complexity. Many existing studies [24,25,26] of the aggregate structural bodies have shown that 2D models can achieve better results with savings in simulation time.

### 3.3. Simulation Plan

In this section, the effect of different aggregates and *a* values on the mechanical properties of the aggregate structures are discussed. There are five kinds of aggregates, including 13.2~19 mm (D1), 9.5~13.2 mm (D2), 4.75~9.5 (D3), 2.36~4.75 mm (D4) and 1.18~2.36 mm (D5). In addition, the *a* value also includes five kinds, which are 0, 0.2, 0.4, 0.6 and 0.8. The simulation plan is as follows:

Step 1: Denote the numerical model of D1 mixing as P1 and record the corresponding aggregate interlocking force and contact force.

Step 2: Obtain the aggregate interlocking force and contact force of the aggregate structure mixed with different proportions of P1 and D2; determine the optimal ratio of P1 and D2 by following the principle of maximum aggregate interlocking force and denote the above ratio of P1 and D2 as P2.

Step 3: Denote the optimal ratio of P2 and D3 as P3 by following the above principle and record the corresponding force.

Step 4: By analogy, complete the design of P4 and P5 and record the corresponding results.

## 4. Results and Discussion

### 4.1. Aggregate Interlocking Forces

**Figure 9:**
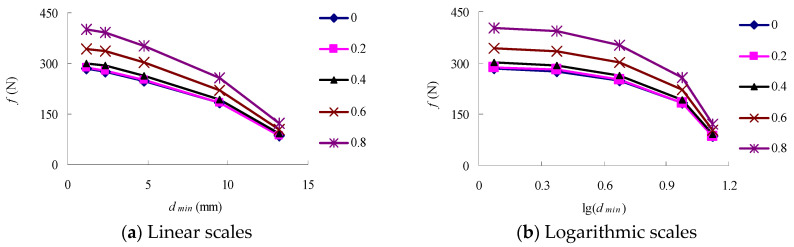
The relationship between *f* and *d_min_*.

**Figure 10:**
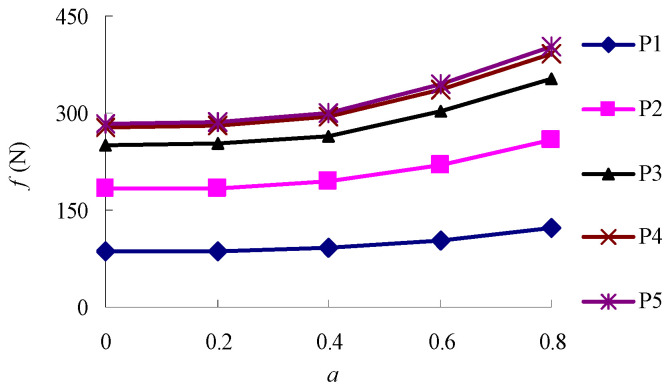
The relationship between *f* and *a*.

The relationship between the aggregate interlocking force (*f*) of P1~P5 and the particle size (*d_min_*) of the minimum-size aggregate in the aggregate structure is shown in Figure 9. Moreover, Figure 10 shows the relationship between values *f* and *a*.

As shown in Figure 9, the aggregate interlocking force increases linearly with the incorporation of D1~D3, and the average of the extruding force of P3 is 2.6 times that of P1. However, with the addition of D4 and D5, the increasing trend of the aggregate interlocking force decreases gradually. The average extruding force of P5 is only 1.33 times and 1.04 times of that of P3 and P4. This indicates that D1~D3 is the main part of the aggregate structure, D4 is the secondary structure of the aggregate structure and plays a role in supporting the main structure, while D5 has little influence on the aggregate interlocking force.

Hence, although the aggregate interlocking force contributed by D4 is lower than that contributed by D1 + D2 + D3, it still accounts for 24.8% of the total aggregate interlocking force. On the other hand, with the addition of D5, the extruding force of the aggregate structure almost no longer increases. The aggregate extruding force contributed by D5 is only 3.6% of the total extruding force, indicating that D5 has little effect on the formation of aggregate skeleton. The above results can provide a basis for dividing coarse and fine aggregates: 2.36 mm is a suitable dividing point. When the aggregate particle size is greater than 2.36 mm, it mainly plays the role of building the main body and secondary skeleton. When the aggregate particle size is less than 2.36 mm, its contribution to skeleton formation is limited.

As shown in Figure 10, the aggregate interlocking force increases with the increase in *a* value, which indicates that the roughness of the aggregate can increase the strength of the aggregate structure. In addition, the contribution of D1~D3 to the strength increase of the aggregate structure increases with the increase in aggregate roughness, but the change in D4 and D5 is not significant.

### 4.2. Aggregate Contact Forces

The aggregate contact forces play an important role in the strength of granular media. In this section, the distribution and number of aggregate contact forces in the numerical model of aggregate structure are studied when the aggregate structure reaches the ultimate load strength. The aggregate contact force is divided into three levels [27]: weak (0, <{*F*}), slightly strong ({*F*}, double {*F*}), strong (double {*F*}, +∞), where {*F*} is the average value of the aggregate contact force.

Table 3 shows the number and distribution of aggregate contact forces for P3, P4 and P5 (*a* = 0.6). Table 4 shows the number and distribution of aggregate contact forces using P5 with different *a* values. The distribution probability of aggregate contact force [*P*(*f*)] is given by Equation (13).

(13) $$P(f)=\frac{N_{i}}{N_{t}}\times 100\%$$

As shown in Table 3, the strong and slightly strong contact forces of the aggregate structure increase continuously with the addition of D4, while the growth slows down. However, the distribution probability of strong and slightly strong contact forces decrease with the sharply increase in the number of weak contact force. This indicates that D4 converts part of the slightly strong contact force composed of D1~D3 into strong contact force and provides additional slightly strong contact force. Moreover, this also explains why the strength of the aggregate structure increases with the increase in D4, but the strengthening trend begins to decline.

With the addition of D5, the number of weak contact forces in the aggregate structure increases sharply, while the number of strong and slightly strong contact forces increases very slowly. This leads to a decrease in the distribution probability of strong and slightly strong contact forces, while the distribution probability of weak contact force increases obviously. Furthermore, it can be seen from the above research that P5 and P4 have little difference in aggregate interlocking forces. This indicates that the strong and slightly strong contact forces are mainly provided by D1~D4. The weak contact force has little effect on the strength enhancement of aggregate structure. The D5 type mainly provides weak contact force for the aggregate structure but makes little contribution to strong and slightly strong contact forces. This explains why the interlocking force of the aggregate structure does not increase obviously with the addition of D5. Therefore, the formation and evolution mechanism of aggregate structure can be regarded as a process of the formation of strong and slightly strong contact forces and the transformation from slightly strong contact force to strong contact force.

Table 4 shows the total number of contact force increases with the increase in *a* value, which indicates that the particle roughness can increase the degree of interlocking of the aggregate. In addition, *P*(*f*) and *N_i_* of the strong contact force and *N_i_* of the slightly strong contact force increase with the increase in *a* value, but *P*(*f*) of the slightly strong and weak contact force decreases with the increase in *a* value. This shows that the increase in the aggregate roughness promotes the transformation from slightly strong and weak contact forces to strong contact force and slightly strong contact force, thus increasing the interlocking forces of the aggregate structure.

### 4.3. Aggregate Structure Composition

**Figure 11:**
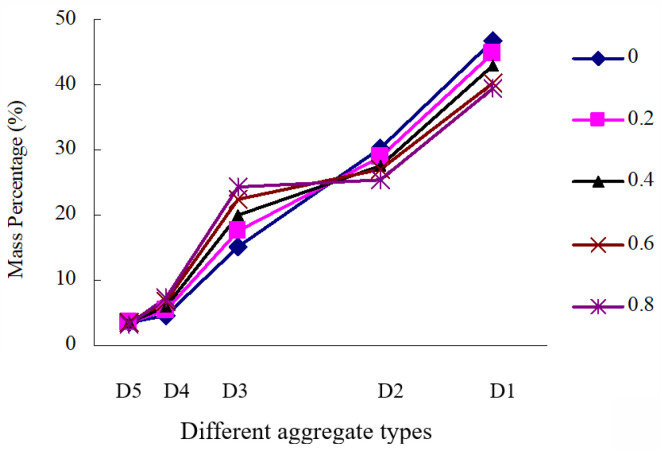
The optimal mass percentage of different types of aggregate.

**Figure 12:**
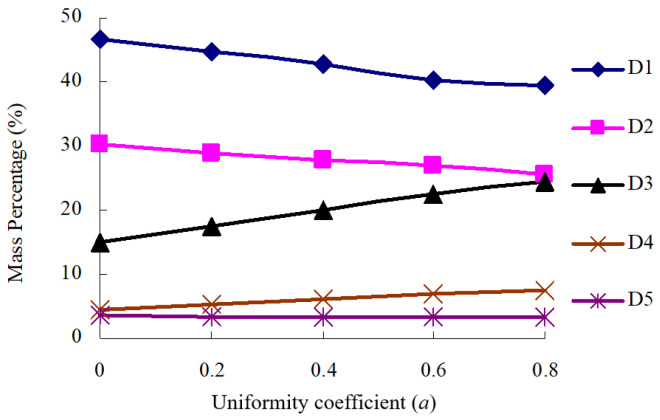
The relationship between *a* value and optimal mass percentage of aggregate.

Figure 11 and Figure 12 show that the optimal mass percentage relationship of D1~D5 in P5 and the relationship between *a* value and optimal mass percentage, respectively.

As shown in Figure 11 and Figure 12, the optional mass percentage of D1, D2, D3, D4 and D5 are 39.48~46.72%, 25.46~30.15%, 15.08~24.38%, 4.58~7.41% and 3.27~3.48% respectively. It can be found that D1 + D2 + D3 accounts for 89.32~91.94% of the total mass of aggregate structure, and there is no significant difference in the total mass percentage of D1 + D2 + D3 under different coarse particles. Moreover, the optimal content of D1 and D2 decreases with the increase in particle roughness, while the optimal content of D3 is the opposite. This is due to the fact that the main aggregate structure composed of it is relatively stable, while it is little affected by the other types of the aggregate.

## 5. The Effect of Ratio of Coarse–Fine Aggregate (CF)

The ratio of CF aggregate (including mineral powder) is determined to be 90:10, 85:15, 80:20, 75:25, 70:30, 65:35 and 60:40. It should be noted that since real aggregate is generally rarely round or round-like, the *a* values of 0.4, 0.6 and 0.8 are used in the calculation of coarse aggregate gradation. Hence, the calculation table of coarse aggregate is shown in Table 5, and the proposed gradation is shown in Table 6.

The performance of the asphalt mixture with different CF ratios is shown in Table 7.

According to Table 7, it can be concluded that the asphalt mixture has good road performance when the CF ratio is 80:20 and 75:25. Therefore, the above proportions can be determined as the final ratios of CF.

## 6. Asphalt Mixture Gradation Optimization and Test Verification

The grading range determined based on the previous test results can be seen in Table 8. The standard gradation (SG) and recommended gradation (RG) are used to compare the performance of the asphalt mixture.

Based on Table 8, it can be seen that the recommended gradation with the “two more and one less” characteristic, which means more coarse aggregate and mineral powder content and less fine aggregate content. This indicates that the structure of coarse aggregate and mineral powder significantly affect the performance of the asphalt mixture and the mortar, respectively, while the impact of fine aggregate on both is not significant. At the same time, compared with the standard gradation, the recommended gradation has a narrower gradation range, which makes it possible to control the aggregate ratio more strictly in the gradation design.

In order to compare the performance indicators of SG and RG, the results of the rutting test(dynamic stability) and SCB test(fracture toughness) are used to detect the high and low temperature performance of the asphalt mixture, as shown in Table 9.

As shown in Table 9, it can be seen that compared with the SG, in the asphalt mixture using the RG, the dynamic stability increased by 30%, and the fracture toughness increased by 26%, which indicates a better quality of use and proves the superiority of the proposed grade in this study.

Although some useful conclusions and findings can be obtained from the numerical simulation study at the two-dimensional scale, the performance pattern of the asphalt mixture will definitely be affected by the three-dimensional scale, so the subsequent study will strive to expand from two-dimensional scale to three-dimensional scale.

## 7. Conclusions

In this study, the mechanical properties of aggregate structures, pavement properties of asphalt mortar and the effect of CF ratio on material properties are studied by means of numerical simulation and laboratory test, respectively. A new gradation based on meso-structure optimization is formed and then verified by laboratory tests. The main conclusions are as follows:

- The coarse aggregate structure can be regarded as a contact force transmission system composed of some strong and sub-strong contact forces. Moreover, the formation–evolution mechanism of coarse aggregate structure can be regarded as a process of the formation of strong and sub-strong contact forces and the transformation from sub-strong contact force to strong contact force.
- The main body of coarse aggregate consists of 4.75~19 mm aggregate, which is composed of a relatively stable structural system and is less affected by other sizes of coarse aggregate. The 2.36~4.75 mm aggregate acts as the secondary structure of the aggregate structure body, which plays the role of supporting the main structure and, at the same time, induces the secondary strong contact force provided by 4.75~19 mm aggregate to transform it into strong contact force and provide additional secondary strong contact force.
- The weak contact force is mainly provided by the aggregates with the particle size less than 2.36 mm, indicating that this aggregate contributes slightly to the formation of coarse aggregate structural bodies. From the point of view of the contribution to the formation of the aggregate structure body, 2.36 mm can be used as the dividing point of coarse and fine aggregates.
- The strength of the aggregate structure increases with the increase in the roughness of the aggregate, which is due to the transformation of sub-strong and weak contact forces to strong and sub-strong contact forces.
- In the asphalt mixture with the recommended gradation, dynamic stability increases by 30%, and fracture toughness increases by 26%, when compared to the mixtures with the standard gradation.
- The effects of other aggregate (recycled aggregate) properties and different temperatures on the aggregates are considered in our subsequent studies.

## Figures and Tables

**Table 1:** Technical properties of asphalt.

Test Project		Results	Technical Specifications
Needle penetration (25 °C, 100 g, 5 s)/0.1 mm		65.2	60~80
Softening point (Universal method)/°C		51.9	≥45
Ductility(5 cm/min, 10 °C)/cm		21.4	≥15
Needle penetration index		−0.818	−1.5~+1.0
60 °C power viscosity/(/Pa·s)		411	≥180
Density (25 °C)/(g/cm^3^)		1.01	-
Wax content (%)		1.55	<2.2
After RTFOT	Mass loss (%)	0.33	−1%~1
	Needle penetration ratio (%)	62.1	≥61
	Ductility (5 cm/min, 10 °C)/cm	6.71	≥6

**Table 2:** Technical properties of aggregates.

Properties	Index		Requirements	Test Value	Test Method
Coarse aggregate	Robustness (%)		≤12	7.7	T 0314
	Stone crushing value (%)		≤26	12.1	T 0316
	Los Angeles abrasion loss (%)		≤28	14.4	T 0317
	Water absorption rate (%)		≤2.0	1.2	T 0304
Fine aggregate	Angularity		≥30	45	T 0345
	sand equivalent (%)		≥60	79	T 0334
	Robustness (%)		≥12	15.4	T 0340
Mineral powder	Hydrophilic coefficient		<1	0.51	T 0353
	Moisture content (%)		≤1	0.44	T 0103
	Particle size (%)	<0.6 mm	100	100	T 0351
		<0.15 mm	90~100	94.3	
		<0.075 mm	75~100	86.7	

**Table 3:** The aggregate contact force of different aggregate structures (*a* = 0.6).

Packing	Index	Grade 1: (Double {*F*}, +∞)	Grade 2: ({*F*}, Double {*F*})	Grade 3: (0, <{*F*})
P3	*P*(*f*)/%	17.97	36.94	45.09
	*N_i_*	108	222	271
P4	*P*(*f*)/%	14.04	33.24	52.72
	*N_i_*	155	367	582
P5	*P*(*f*)/%	9.93	24.17	65.87
	*N_i_*	162	393	1071

**Table 4:** The aggregate contact force of P5 with different *a* values.

*a*	Index	Grade 1: (Double {*F*}, +∞)	Grade 2: ({*F*}, Double {*F*})	Grade 3: (0, <{*F*})
0	*P*(*f*)/%	7.13	21.57	71.30
	*N_i_*	119	360	1190
0.2	*P*(*f*)/%	7.73	22.69	69.59
	*N_i_*	127	373	1144
0.4	*P*(*f*)/%	8.62	23.51	67.87
	*N_i_*	139	379	1094
0.6	*P*(*f*)/%	9.93	24.17	65.87
	*N_i_*	162	393	1071
0.8	*P*(*f*)/%	11.65	24.49	63.85
	*N_i_*	196	412	1074

**Table 5:** Coarse aggregate calculation.

*a*	D1:D2:D3:D4	Coarse Aggregate Ratio	Sieve Passing Rate (%)				
			19 mm	13.2 mm	9.5 mm	4.75 mm	2.36 mm
0.4	42.30:27.64:20.00:6.08	60~90	100	60.4~73.6	34.5~56.3	15.7~43.8	10~40
0.6	40.40:26.93:22.44:6.82		100	62.4~74.9	37.3~58.2	16.4~44.2	10~40
0.8	39.48:25.46:24.38:7.41		100	63.3~75.5	39.6~59.7	16.9~44.6	10~40

**Table 6:** Initial proposed gradation.

CF Aggregate Ratio	Sieve Passing Rate (%)									
	19 mm	13.2 mm	9.5 mm	4.75 mm	2.36 mm	1.18 mm	0.6 mm	0.3 mm	0.15 mm	0.075 mm
90:10	100	62.0	37.1	16.3	10	7.30	5.58	4.43	3.68	3.24
85:15	100	64.1	40.6	21.0	15	10.96	8.38	6.65	5.53	4.86
80:20	100	66.2	44.1	25.6	20	14.61	11.17	8.87	7.37	6.48
75:25	100	68.3	47.6	30.3	25	18.26	13.96	11.08	9.21	8.11
70:30	100	70.5	51.1	34.9	30	21.91	16.75	13.30	11.05	9.73
65:35	100	72.6	54.6	40.0	35	25.56	19.55	15.52	12.89	11.35
60:40	100	74.7	58.1	44.2	40	29.22	22.34	17.73	14.74	12.97

**Table 7:** Test results.

CF Ratio	Dynamic Stability	Fracture Toughness
90:10	848	0.94
85:15	1190	1.14
80:20	1336	1.26
75:25	1446	1.41
70:30	1125	1.09
65:35	834	0.79
60:40	768	0.88

**Table 8:** Recommended gradation range.

Gradation	Sieve Passing Rate (%)									
	19 mm	13.2 mm	9.5 mm	4.75 mm	2.36 mm	1.18 mm	0.6 mm	0.3 mm	0.15 mm	0.075 mm
RG	100	64~70	42~49	25~31	20~25	14~18	11~14	9~12	7~10	6~9
SG	100	70~92	60~80	34~62	20~48	13~36	9~26	7~18	5~14	4~8

**Table 9:** Road performance of asphalt mixture.

Technical Properties	RG	SG	Performance Ratio
Dynamic stability (KN)	3187	2451	1.30
Fracture toughness (Mpa·m^0.5^)	1.122	0.893	1.26

## Data Availability

The data presented in this study are available in the article.

## Abbreviations

*S_i_*(*i*:1,2,3)	two-dimensional mapping area of the *i*th type coarse aggregate
*m_i_*	coarse aggregate mass of the *i*th type
*W*	particle formation area width
*ρ_i_*	apparent density of the *i*th type coarse aggregate
*G*	shear modulus
*U*	amount of overlap between particle units
*f_n_*	contact force in each direction
Ε	Poisson’s ratio
*v^t^*^+1^/*v^t^*	target wall speed at time step *t* + 1 and *t*
Δ*σ*	difference between the current pressure and the target pressure
*A*	relaxation factor
*L*	the length of target wall
*k_n_*	average stiffness
*N*	total number of contacts between the target wall and particles
*N_i_*	aggregate contact force number of grade “*i*”
*N_t_*	total number of aggregate contact forces
*R*	average radius of contact particles
*R_it_*/*r_it_*	radius of particle and small particle at the *i*th type coarse aggregate
*X_it_*/*Y_it_*	X and Y coordinates of particle at the *i*th type coarse aggregate
*a*	uniformity coefficient
